# Repression of Mitochondrial Citrate Synthase Genes by Aluminum Stress in Roots of *Secale cereale* and *Brachypodium distachyon*

**DOI:** 10.3389/fpls.2022.832981

**Published:** 2022-04-07

**Authors:** Diaa Abd El-Moneim, Roberto Contreras, Javier Silva-Navas, Francisco Javier Gallego, Ana M. Figueiras, Cesar Benito

**Affiliations:** Department of Genetics, Faculty of Biology, Complutense University of Madrid, Madrid, Spain

**Keywords:** aluminum tolerance, mitochondrial citrate synthase, expression changes, *Secale cereale*, *Brachypodium distachyon*

## Abstract

Aluminum (Al) toxicity in acid soils influences plant development and yield. Almost 50% of arable land is acidic. Plants have evolved a variety of tolerance mechanisms for Al. In response to the presence of Al, various species exudate citrate from their roots. Rye (*Secale cereale* L.) secretes both citrate and malate, making it one of the most Al-tolerant cereal crops. However, no research has been done on the role of the mitochondrial *citrate synthase* (*mCS*) gene in Al-induced stress in the rye. We have isolated an *mCS* gene, encoding a mitochondrial CS isozyme, in two *S. cereale* cultivars (Al-tolerant cv. Ailés and Al-sensitive inbred rye line Riodeva; *ScCS4* gene) and in two *Brachypodium distachyon* lines (Al-tolerant ABR8 line and Al-sensitive ABR1 line; *BdCS4* gene). Both *mCS4* genes have 19 exons and 18 introns. The *ScCS4* gene was located on the *6R*L rye chromosome arm. Phylogenetic studies using cDNA and protein sequences have shown that the *ScCS4* gene and their ScCS protein are orthologous to *mCS* genes and CS proteins of different Poaceae plants. Expression studies of the *ScCS4* and *BdSC4* genes show that the amount of their corresponding mRNAs in the roots is higher than that in the leaves and that the amounts of mRNAs in plants treated and not treated with Al were higher in the Al-tolerant lines than that in the Al-sensitive lines of both species. In addition, the levels of *ScCS4* and *BdCS4* mRNAs were reduced in response to Al (repressive behavior) in the roots of the tolerant and sensitive lines of *S. cereale* and *B. distachyon*.

## Introduction

The most common metal in the Earth’s crust is aluminum (Al). In non-acid soils, Al is harmless; but in acidic soils, Al is solubilized into the toxic Al^3+^ cation (Ritchie, 1995), which inhibits root elongation (Panda et al., 2009). Internal and external mechanisms have developed in plants to deal with Al^3+^. The exudation of organic anions from root apices is an exclusion mechanism identified for many species with strong genetic support (e.g., malate, citrate, and, in some species, oxalate) (Mariano and Keltjens, 2001). Two distinct patterns of Al-induced organic acid secretion have been identified. The release of organic acids starts immediately after the addition of Al in pattern I (Hede et al., 2001; Ma et al., 2001). The activation of the anion channel was involved in this pattern. In pattern II, the release of organic acids begins after a marked lag phase (Hede et al., 2001; Ma et al., 2001). The genes of organic acids metabolism are implicated in this pattern. The ability of different plants to withstand toxic concentrations of Al^3+^ varies greatly. In response to Al stress, the rye *S. cereale*, one of the most Al-tolerant cereal crops, secretes citrate and malate from its roots (Li et al., 2000). Al-stress tolerant cultivars (Ailés and Petkus) and the sensitive Riodeva inbred line show pattern II (inducible) of malic exudation (Abd El-Moneim et al., 2014, 2015; Sánchez-Parra et al., 2015). For citrate, the tolerant Petkus also showed an inducible exudation pattern (Abd El-Moneim et al., 2014). According to the results of Ma et al. (2001), however, the sensitive Riodeva does not show such an inducible pattern (Abd El-Moneim et al., 2014). The model grass *B. distachyon* also secretes both organic acids following an inducible exudation pattern (pattern II) (Contreras et al., 2014). Tolerance genes for Al-activated malate transporter (*ALMT*) and Al-activated citrate transporter (*AACT* or *MATE*) promote organic anion efflux from roots. These genes have been identified in various species for Al tolerance. In many plants, the activities of enzymes involved in citrate metabolism have been investigated (Mariano et al., 2005). One of them is citrate synthase (CS) activity encoded by several genes. In *S. cereale* (Li et al., 2000), *Phaseolus vulgaris* (Mugai et al., 2000), and *Cassia tora* (Yang et al., 2004), exposure to Al increased CS root activities, but not in wheat (*Triticum aestivum*) or triticale (Yang et al., 2004). In soybean (*Glycine max*), Al treatment enhanced mitochondrial CS enzyme activity and their gene expression (Eticha et al., 2010). On the contrary, in *Coffea arabica*, CS activities decrease after Al shock (Ramírez-Benítez et al., 2008). Finally, CS activities in the tolerant and sensitive triticale lines and their response to pH in the presence or absence of Al were similar (Hayes and Ma, 2003). In this work, the authors propose that the exudation of organic acids is not very related to their metabolism. In addition to soybean, variations in *CS* gene expression have also been examined in *P. vulgaris* and *Arabidopsis thaliana*. In *P. vulgaris*, no major differences in *CS* expression were found (Eticha et al., 2010). Transcriptomic analysis of *A. thaliana* root responses to Al revealed no significant enhancement in *CS* transcript abundance (Kumari et al., 2008). In cultured carrot (*Daucus carota*) cells (Koyama et al., 1999), *A. thaliana* (Koyama et al., 2000), and canola (*Brassica napus*) (Anoop et al., 2003) plants, overexpression of mitochondrial *CS* genes (*mCS*) resulted in enhanced citrate efflux. There is an essential controversy over whether *CS* overexpression results in the increase in citrate exudation and greater tolerance to Al stress in tobacco and alfalfa transgenic plants (de la Fuente et al., 1997; Delhaize et al., 2001; Osawa and Kojima, 2006; Barone et al., 2008; Han et al., 2009). The overexpression of a bacterial *CS* gene in tobacco and papaya transgenic plants improved Al tolerance and citrate overproduction (de la Fuente et al., 1997). Delhaize et al. (2003) concluded that internal citrate concentrations and citrate efflux in tobacco are highly insensitive to large changes in either mitochondrial CS activity or cytosolic isocitrate dehydrogenase (IDH) activity and suggested other factors such as transport out of the roots or control citrate efflux. The findings obtained by Osawa and Kojima (2006) indicated that Al-inducible expression of *mCS* coupled with enhanced citrate release mediates Al resistance in the tree *Paraserianthes falcataria*. Despite the wealth of data on *mCS* gene activity reported in other plant species, less is known on their responsiveness to Al stress in *S. cereale* and *B. distachyon*.

This study aimed to isolate the gene, identify the chromosomal location, analyze the molecular variability, and observe the changes in the expression of mitochondrial *citrate synthase* gene (*mCS*) by Al stress in *S. cereale* and *B. distachyon*.

## Materials and Methods

### Plant Materials

Six different cultivars were used in the experiments (four *S. cereale* cultivars and two *B. distachyon* cultivars). The tolerance or sensitivity of the *S. cereale* cultivars and of the lines of *B. distachyon* to Al was established by root growth tests in the absence of and in the presence of Al at different concentrations in previous studies carried out by our research group (Contreras et al., 2014; Abd El-Moneim et al., 2015). Table 1 shows the Al tolerance for the studied cultivars. In these previous works, we also estimated the exudation by the malate and citrate roots in the presence and absence of Al. Chromosomal location of the *ScCS* gene was identified in rye (Imperial cultivar), hexaploid wheat *T. aestivum* (Chinese Spring cultivar), their corresponding wheat-rye amphiploid (Chinese Spring-Imperial), seven wheat-rye disomic addition lines (*1R* to *7R*), and the ditelosomic wheat-rye addition line *6R*S.

**TABLE 1:** Names of the studied rye and *Brachypodium* cultivars and their response to aluminum stress.

Species	Cultivar	Response to aluminum	References
***S. cereale***	Ailés	Tolerant	Fontecha et al., 2007
***S. cereale***	Imperial	Tolerant	Abd El-Moneim et al., 2015
***S. cereale***	Petkus	Tolerant	Abd El-Moneim et al., 2015
***S. cereale***	Riodeva (inbred line)	Sensitive	Fontecha et al., 2007
***B. distachyon***	ABR8	Tolerant	Contreras et al., 2014
***B. distachyon***	ABR1	Sensitive	Contreras et al., 2014

### Cloning of *ScCS4* Gene From Rye

Seven-day-old seedlings were subjected to a nutrient solution containing Al in the form of AlK (SO4)_2_⋅12H_2_O (pH 4.0) at a concentration of 150 μM for 24 h to isolate the *ScCS4* gene of rye. Root samples were frozen in liquid nitrogen and held at −80°C until required. Using TRIzol Kit (Invitrogen), RNA was obtained from the roots and then converted to cDNA using High-Capacity cDNA Reverse Transcription Package (Applied Biosystems). From the sequence of the *HvCS4* gene (reference AK248736.1) in barley, one pair of primers was designed to amplify *ScCS4* cDNA in the rye by PCR (Table 2, *ScCS4*-cDNA–F and *ScCS4*-cDNA-R).

**TABLE 2:** Sequences of the primers used in this work, annealing temperature (Tm) and number of cycles used in the PCR.

Primer	Amplified introns	Sequence (5′ → 3′)	Tm°C	Cycles	References
ScCS4-cDNA-F		GCAGCGTGCTCCGGCCATGGCGTT	55	35	Sato et al., 2009
ScCS4-cDNA-R		TTGGTAAGACGATGAACTGGTGTA			Sato et al., 2009
ScCS4-IN1-2-F	1 and 2	CTCGAGGTTGCGATCCCGTATGG	58	35	This research
ScCS4-IN1-2-R		CCTGCAGCTGGGACCTAAGATCA			
ScCS4-IN3-F	3	GGAAATGATTCCGGAGCAACAGG	58	35	This research
ScCS4-IN3-R		CTCTGACTTTAGTTTCTTCAAGC			
ScCS4-IN4-5-F	4 and 5	GGAAACATAACTGTGGACATGGT	58	35	This research
ScCS4-IN4-5-R		AGAGAGACCTCTAAATCGAATAC			
ScCS4-IN6-F	6	TGGCTTCTTTTGACGGGAAAGGT	58	35	This research
ScCS4-IN6-R		CATCAACTTGCTCCTTGGTTGGC			
ScCS4-IN7-F	7	TTAGCCGTTCGACTGTTCCAGGT	58	35	This research
ScCS4-IN7-R		GCATCTATCGCCTTATAGACATA			
ScCS4-IN8-9-10-11-F	8, 9, 10, and 11	CTCATCCAATGACACAGTTTACC	58	35	This research
ScCS4-IN8-9-10-11-R		ACCAGATGCCCTGTATGAGCACT			
ScCS4-IN12-F	12	AGTGCTCATACAGGGCATCTGGT	58	35	This research
ScCS4-IN12-R		CAGTGCCGCTGCAAAAGAAAGGT			
ScCS4-IN13-14-F	13 and 14	TGGAAGTGCTCTGTCAGATCCCT	58	35	This research
ScCS4-IN13-14-R		CTCCATGACCATAGCCAGGAACA			
ScCS4-IN15-16-17-18-F	15, 16, 17, and 18	GAGGACCCACTTTTCCAACTGGT	58	35	This research
ScCS4-IN15-16-17-18-R		CCAAGGGCACGGTCCCAGATGAG			
ScCS4-loc-F		GGATGACTGGAATGCTTTGGGAG	58	33	This research
ScCS4-loc-R		TTGTTTTGGGCATTCCCTTGTCA			
ScCS4-QPCR-F		CTCGTGCCAAAGGGAGTTTG	60		This research
ScCS4-QPCR-R		ACTCGGTGAGGATCGGAGG			
18S4-FWD		TCAACGAGGAATGCCTAGTAAGC	60		Sánchez-Parra et al., 2015
18S4-REV		ACAAAGGGCAGGGACGTAGTC			

The PCR was performed using 20 μl of the reaction mixture containing 30 ng of DNA, 5 pmols of each primer, and 10 μl of Taq PCR Master Mix (Qiagen). Amplifications were carried out in the thermal cyclers PTC-100 (MJ Research) with the following program: an initial step of 3 min at 94°C, 35 cycles of 1 min at 94°C, 1 min at 55°C, and 2 min at 72°C followed by a final extension step of 7 min at 72°C. PCR products were visualized on 1.5–2% TAE agarose gels.

**FIGURE 1:**
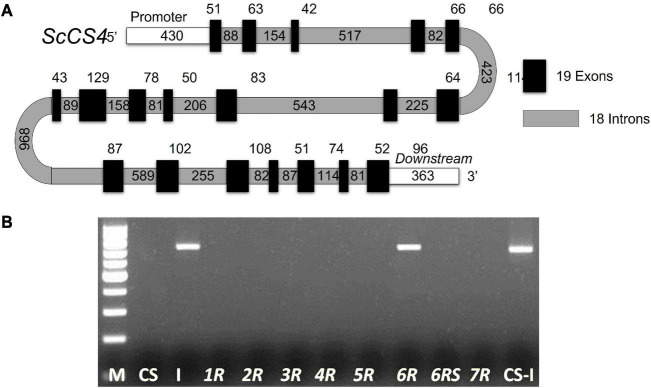
**(A)** Schematic representation of the *ScCS4* gene. This gene has 19 exons and 18 introns. The exons are depicted as black boxes, and the introns are depicted as gray boxes. The numbers indicate their respective sizes in bp. A 430-bp fragment corresponding to the upstream (promoter) region and a 363-bp fragment corresponding to the downstream region were isolated. **(B)** Chromosomal location of the *ScCS4* gene. **CS:** Chinese Spring (*T. aestivum*). **I:** Imperial (*S. cereale*). **CS-I**: Wheat–rye amphiploid. ***1R***, ***2R***, ***3R***, ***4R***, ***5R***, ***6R***, and ***7R***: Wheat-rye disomic addition lines (Chinese Spring–Imperial). ***6R*S**: rye chromosome arm *6R*S ditelosomic addition line. **M**: molecular marker.

The PCR product was cloned into the TOPO TA Cloning Kit according to the conditions specified by the supplier (Invitrogen). The sequences were identified *via* an ABI PRISM 3700 Genetic Analyzer. Nine pairs of primers (from *ScCS4*-IN1-2-F to *ScCS4*-IN15-16-17-18-R) were designed on successive exons (Figure 1A) to amplify different introns to obtain the genomic sequence of the *ScCS4* gene (Table 2). The PCR mixture was the same as described previously but nine pairs of primers were designed to amplify the introns. The PCR program used was also the same as described previously but the annealing temperature was 58^°^C (Table 2).

The secondary structures of the hypothetical rye proteins were obtained using the PSIPRED protein structure prediction server,^1^ while the SWISS-MODEL program was used to create a three-dimensional model of the hypothetical rye ScCS protein. In this regard, Jmol software^2^ was used to generate a protein model for *ScCS* protein (cultivar Petkus).

### Chromosome Arm Assignment of *ScCS4* Gene

Two pairs of primers (Table 2, ScCS4-loc-F, and ScCS4-loc-F) were designed based on the sequences of *ScCS4* of Imperial rye cultivar and *TaCS4* of *T. aestivum* to locate the *ScCS4* gene. This pair of primers was developed just to amplify in Imperial rye but not in wheat. The PCR conditions (reaction mix and program) were the same as described previously but with 33 cycles and 58^°^C annealing temperature (Table 2). To locate the *ScCS4* gene, gDNAs of wheat-rye disomic and ditelosomic lines were extracted from young leaves with the DNeasy Plant Mini Kit (Qiagen).

### Genetic Diversity and Phylogenetic Relationships Analysis

Different parameters (NS: number of sites, INDEL: insertion-deletion polymorphism, IMS: invariable monomorphic sites, VPS: variable polymorphic sites, SVS: singleton variable sites, PIS: parsimony-informative sites, CS: sequence conservation, SC: synonymous changes, NSC: non-synonymous changes; Supplementary Table 1) were computed to describe the genetic diversity for *ScCS4* among rye cultivars and some Poaceae species using DnaSP software v5.

In addition, using MEGA X software (Tamura et al., 2013), phylogenetic relationships between several CS proteins from Poaceae and other eudicot species were studied using different approaches, such as the amino acid substitution model and the neighbor-joining clustering method. The dendrogram’s robustness was examined using bootstraps with 10,000 replicates.

### RNA Extraction and Quantitative RT-PCR

For expression analyses, 30 seeds were always germinated and grown per treatment. The seeds were sterilized with HgCl_2_ (0.1%) for 10 min, washed several times with de-ionized water, and incubated overnight on moist filter paper in the dark at 4°C in Petri dishes. Later, the seeds were incubated for two more days in the dark at 20°C in Petri dishes. Germinated seeds were placed on a nylon mesh floating on a continuously aerated nutrient solution containing 0.4 mm CaCl_2_, 0.65 mm KNO_3_, 0.25 mm MgCl_2_⋅6H_2_O, 0.01 mm (NH_4_)_2_SO_4_, and 0.04 mm NH_4_NO_3_ (pH 4.0). Containers with this aerated nutrient solution were placed in a growth chamber at 20°C under 16-h day illumination, a light intensity of 12 W/m^2^, and 60% of humidity (Abd El-Moneim et al., 2015). The seedlings of the cv. Petkus and the Riodeva inbred line of *S. cereale* were exposed to five different nutrient solutions containing 0, 25, 50, 100, and 300 μM AlK (SO_4_)_2_, respectively. Meanwhile, the same number of seedlings were exposed to 300 μM AlK (SO_4_)_2_ at different times (0, 4, 8, 12, and 24 h). In the case of ABR1 and ABR8 cultivars of *B. distachyon*, the seedlings were exposed to a nutrient solution containing 0 and 20 μM AlK (SO_4_)_2_ for 24 h. Following that, the root apices (15 mm) and leaves were excised and frozen in liquid nitrogen. TissueLyser II (Qiagen) and 5-mm Stainless Steel Beads were used to homogenize the samples. Total RNA was extracted from leaves and roots from 30 plants per genotype and exposure time using TRIzol and a PureLink RNA Mini Kit. A NanoDrop ND-1000 spectrophotometer was used to determine the quality of the RNA. A High-Capacity cDNA Reverse Transcription Kit (Applied Biosystems) was used to reverse transcript (2 μg) the total RNA. Depending on cDNA sequences from the previously isolated *ScCS4* gene and the DOE-JGI genome sequence for *BdCS4* gene of *B. distachyon* accession Bd21, specific primers were designed with Oligo software (Table 2, ScCS4-QPCR-F, and ScCS4-QPCR-R). Furthermore, the *18S* gene’s mRNA (Table 2, 18S-FWD, and 18S-Rev) was used as a housekeeping gene. The qRT-PCR was performed on an Applied Biosystems 7900 HT fast real-time PCR device under the following conditions: one stage at 95°C for 10 min, followed by 40 cycles of 95°C for 15 s and 60°C for 1 min. All PCR samples and controls were prepared in duplicates. All 96-well plates contained two standard curves (target gene and endogenous control) for the use of the “relative standard curve approach” for data analysis. Unknown sample values are interpolated from the standard curves in this method. To assure that PCR products were not attributable to amplification of contaminant gDNA, duplicate control reactions without reverse transcription were included for each sample.

## Results

### The Isolation of *ScCS4* Gene of Rye

A 1,419-bp cDNA fragment (including the stop codon) was amplified from the roots of Ailés, Imperial, Petkus, and Riodeva mRNA treated with Al for 24 h using primers of the *HvCS4* gene (Table 2, ScCS4-cDNA–F, and ScCS4-cDNA-R). The rye fragments from Ailés, Imperial, Petkus, and Riodeva were of the same size as that found in barley *HvCS4* gene. Eight samples of rye cDNA fragments were cloned (one from Ailés, four from Imperial, two from Petkus, and one from Riodeva), sequenced, and compared among them (Supplementary Data 1) and with other plant species’ sequences (Supplementary Data 2). These eight cDNA fragments were coded for putative CS enzymes with 472 amino acids (Supplementary Data 1A). These sequences are in the NCBI (GenBank accession numbers from OM718863 to OM718870).

The *ScCS4* rye cDNA sequence of Ailés shared 99% identity with *T. aestivum TaCS4* (Accession No. AK455004.1), 98% identity with *Aegilops tauschii AetCS4* (Accession No. XM020301802.1), 97% identity with barley *HvCS4* cDNA (Accession No. AK248736.1), 94% with *B. distachyon* (Accession No. XM003571152.3), and 88% of *Oryza sativa OsCS4* cDNA (Accession No. XM015762564.1).

The genomic sequence of the *ScCS4* gene from the Ailés cultivar was obtained using nine primer pairs designed in successive exons (Table 2, from ScCS4-IN1-2-F to ScCS4-IN15-16-17-18-R) in order to amplify the corresponding introns. The predicted location of the different rye *ScCS4* introns was described by comparison of the rye cDNA sequence with the rye genomic sequence and with the orthologous *B. distachyon* gene. The *ScCS4* gene displayed a total of 19 exons and 18 introns. Exon and intron sizes are shown in Figure 1A.

### Molecular Diversity of the *ScCS4* Gene in *Secale cereale* and Different Poaceae Species

We analyzed the variability among 19 exons in the *ScCS* gene’s cDNAs using the eight different sequences (different clones) obtained: one from Ailés, four from Imperial, two from Petkus, and one from Riodeva (Supplementary Data 1, 1A). Moreover, we carried out a diversity analysis in each of the 19 exons of *CS4* in rye, comparing to several orthologous exons from other Poaceae and eudicots species (exon sequences of six species of Poaceae) (Supplementary Data 2).

In rye cDNA sequences, only exons 4, 5, 11, 14, 15, 17, and 19 showed SNPs, being exon 17 the most variable (Supplementary Table 1). Twelve SNPs of the *ScCS4* gene were detected; four and two were exclusive for Imperial (clone Imperial 5) and sensitive inbred line (Riodeva), respectively, while one SNP was exclusive for Imperial (clone Imperial 1), Imperial (clone Imperial 4), Petkus (clone Petkus 2), and Petkus (clone Petkus 9) (Supplementary Table 1). Two non-synonymous changes (H62Y and K343R) were observed in the *ScCS4* gene of Riodeva (Supplementary Data 1A). We used the PROVEAN software^3^ to predict whether these changes have an impact on the biological function of the ScCS4 protein. The results obtained indicate that both changes are neutral (prediction cutoff −2.5) (Supplementary Figure 1).

In the comparisons between Poaceae (Supplementary Table 2), exon 9 revealed the highest average SNPs per 100 nucleotides (30). Furthermore, all the exons were variable, the total number of SNPs was 329 (Supplementary Tables 2, 3), and the nucleotide diversity was 0.10097 (Supplementary Table 3). Regarding the changes in the hypothetical proteins encoded by the *ScCS* gene, the number of synonymous changes was higher than that of non-synonymous changes in comparing Poaceae species. However, the situation was reversed in the case of rye sequences, where the number of non-synonymous changes was higher than that of synonymous changes (Supplementary Table 3). Likewise, the degree of conservation between the rye sequences (0.992) was much higher than the degree of conservation between the Poaceae sequences (0.768), as expected for different species. However, the nucleotide diversity among studied rye sequences was 0.00269, lower than the nucleotide diversity (0.10097) among different Poaceae sequences (Supplementary Table 3). It was noted that no insertions or deletions (Indels) were detected among all the studied cDNA sequences of rye as well as among cDNA sequences of Poaceae species.

### Chromosomal Location of the *ScCS4* Gene

A specific band of Imperial (I) rye cultivar was amplified using a *CS4* specific pair of primers (Table 2, ScCS4-loc-F and ScCS4-loc-F) in this cultivar and in the addition line Chinese Spring-Imperial (CS-I) with the chromosome *6R*. However, this band was not observed in the addition line (CS-I) with the chromosome arm *6R*S. Therefore, this gene was located by disomic and ditelosomic wheat-rye addition lines on the chromosome arm *6R*L (Figure 1B) of the Imperial rye.

### Analysis of the Secondary and Tertiary Structures of the ScCS Protein in Rye

The secondary and tertiary structures of the hypothetical rye proteins (Supplementary Data 3) were obtained using PSIPRED v3.0 and SWISS-MODEL programs, respectively. These programs indicated that ScCS4 is a mitochondrial rye protein. The CS domain has 23 α-helix regions with high predictive confidence and 3 β-sheet regions (Supplementary Figure 1); the first observed β-sheet region (in yellow color and without assigned number) has a very low predictive confidence. Only four amino acid substitutions were detected after comparing the four hypothetical rye proteins obtained in this study. However, when comparing them with the barley sequence (AK248736) and rice sequences (*OsCSY5* and *OsCS*), we found 5, 30, and 33 amino acid substitutions, respectively. The SWISS-MODEL software identified (with a resolution of 2.00 Å) an *A. thaliana* CS (6k5v.1.A) as a template for all rye mitochondrial CS sequences (Nishio and Mizushima, 2020). In all cases, the percentage of identity in the amino acid sequence was 83.48%. The predicted hypothetical quaternary structure is a homo-dimer. The generated protein model for ScCS protein (cultivars Ailés and Petkus) is shown in Supplementary Figure 1. Nishio and Mizushima (2020) have described three functionally important residues (His308, His354, and Asp409) in *A. thaliana*. In our rye sequences, the equivalent residues are His307, His353, and Asp408. These residues are conserved in all rye cultivars and Riodeva line. Several disulfide bridge-forming residues and other cysteine residues are conserved in all rye cultivars (Cys 108, Cys209, Cys 365, and Cys 467). The two non-synonymous changes (H62Y and K343R) observed in the Riodeva *ScCS4* protein (Supplementary Data 1A) do not alter the secondary and tertiary structures of the protein. Therefore, these changes probably do not affect the correct functioning of the enzyme.

### Phylogenetic Relationships Among *ScCS4* cDNAs and Amino Acid Sequences and Other CS4 in Poaceae

**FIGURE 2:**
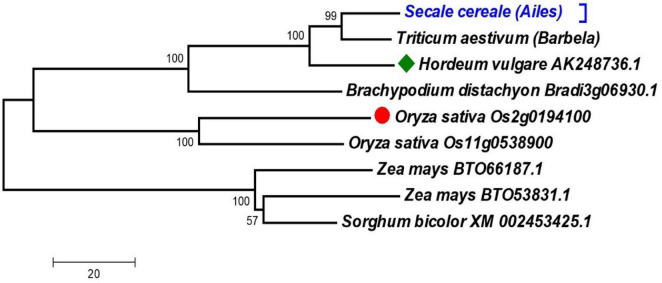
The dendrogram was obtained from the sequences of cDNAs of *ScCS4* gene and *CS* genes of seven different Poaceae species. The studied sequences of cDNAs and proteins were extracted from NCBI and Phytozome. Bootstraps with 10,000 replicates were calculated to test the robustness of the dendrogram. The red circle indicates the starting rice sequence and the green diamond indicates the barley sequence. The *ScCS4* sequence obtained has been indicated with light blue text.

We used two methods to confirm that the isolated rye gene is the ortholog of the mitochondrial CS genes of rice and barley (which were employed to design the primers used to amplify rye sequence). First, we studied the phylogenetic relationships among the cDNAs of *ScCS4* gene and other CS4 Poaceae genes. Second, we studied the phylogenetic relationships among the amino acids of the putative ScCS4 protein and the amino acid sequences of other CS4 Poaceae proteins that are available in the NCBI databases (Supplementary Data 2). To ensure that the dendrogram structure and the different clusters obtained were consistent, different models of nucleotide substitution genetic distances for cDNAs, amino acid substitutions for proteins, and clustering methods were used. All the phylogenetic trees obtained always gave dendrograms with the same structure and very similar bootstrap values. Figure 2 showed the dendrogram obtained using cDNA sequences. The rye sequences are grouped in the same subcluster with those of other Triticines, such as wheat and barley. Maize and sorghum are grouped into another subcluster.

In the same context, Supplementary Figure 2 showed the phylogenetic tree obtained by various CS4 proteins belonging to rye and other Poaceae and eudicots species (Supplementary Data 4). The CS4 proteins were assembled in two clusters, one with the monocot species and the other with the eudicot species. The monocot cluster has two subclusters, one of them containing the rye ScCS4 protein and the CS4 proteins of *T. aestivum* (from the genomes A, B, and D), *T. urartu*, *Ae. tauschii*, *H. vul*gare, *B. distachyon*, and *B. stacei*. The other subcluster groups the CS4 proteins of *O. sativa*, *O. brachyantha*, *Sorghum bicolor*, *Zea mays*, *Panicum virgatum*, *P. hallii*, *Setaria italica*, and *S. viridis*. The eudicots cluster also has two subclusters. One of them contains the Brassicaceae species (*B. napus*, *B. oleracea*, *B. rapa*, *A. thaliana*, *A. lyrata*, *Raphanus sa*tivus, *Eutrema salsugineum, Camelina sativa*, and *Capsella rubella*). The other subcluster contains a Malvaceae species (*Theobroma cacao*), three Rosaceae and Rhamnaceae species (*Prunus sibirica*, *P. persica*, and *Ziziphus jujuba*), and two Cucurbitoidae species (*Cucumis sativus* and *C. melo*).

### Expression Pattern of *ScCS4* and *BdCS4* Genes in Response to Aluminum Treatment

**FIGURE 3:**
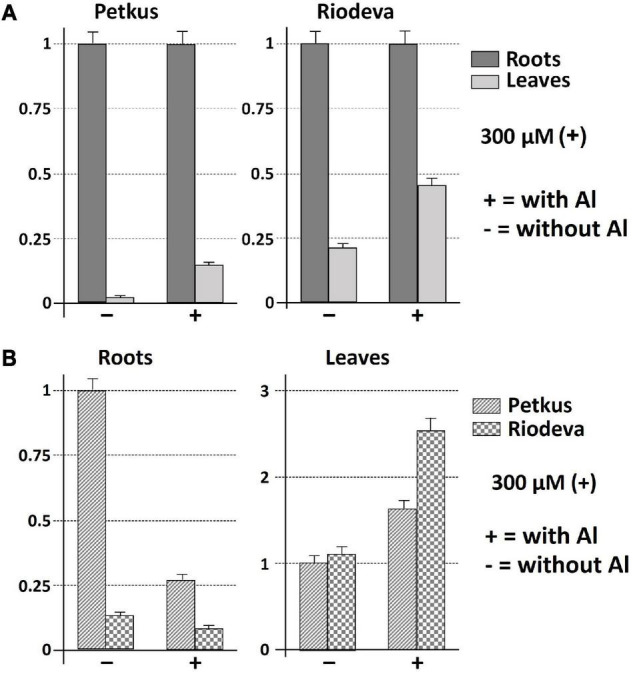
Expression patterns (qRT-PCR) of *ScCS4* under 300 μM AlK (SO4)_2_ at 24 h of treatment with AlK (SO4)_2_ (+) and without AlK (SO4)_2_ (−) using root apices and leafs of Riodeva (sensitive inbred line) and Petkus (tolerant cultivar). The expression level of 18S rRNA was used as a loading control. Error bars represent standard deviations (SDs). All data are the means of three independent biological and technical replicates. **(A)** *ScCS4* expression level of leaves compared with roots in Petkus and Riodeva. The relative expression level 1 for Petkus roots with and without aluminum and the relative expression level 1 for Riodeva roots with and without aluminum do not mean that there is the same amount of citrate synthase messenger RNA in the different situations. In all four cases, the roots have been selected as a reference. **(B)** *ScCS4* expression level of Riodeva compared with Petkus in roots and leaves. Change at each time point is expressed as the relative difference in expression compared with roots of the Petkus without Al or with leaves of the Petkus without Al. Y-axis: change (fold difference) at each time point is expressed as the relative difference in expression compared with the case selected as reference (relative expression level 1).

**FIGURE 4:**
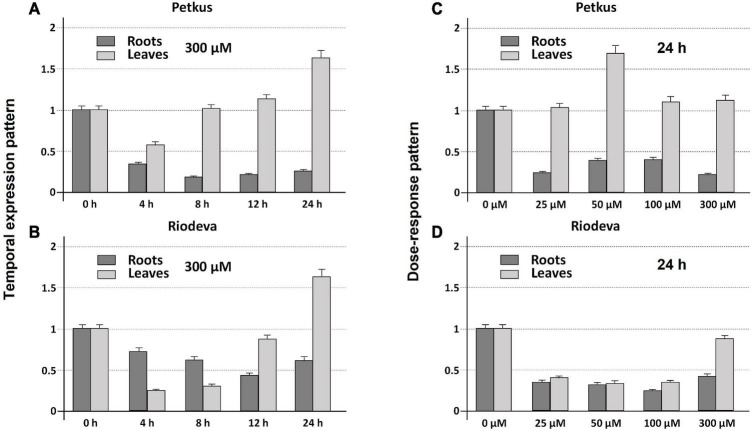
Temporal expression **(A,B)** and dose-response patterns **(C,D)** (qRT-PCR) of root apices and leaf cDNA transcripts of *ScCS4* gene from the Petkus (tolerant cultivar) **(A,C)** and Riodeva (sensitive inbred line) **(B,D)** in the absence of Al (0 h) or with 300 μM of AlK (SO4)_2_ at 4, 8, 12, and 24 h (temporal expression patterns) or with Al at 0, 25, 50, 100, and 300 μM for 24 h (dose–response patterns). Change is expressed as the relative difference in expression without Al (0 h or 0 μM) compared with each time point. The expression level of 18S rRNA was used as a loading control. Error bars represent standard deviations (SDs). All data are the means of three independent biological and technical replicates. Y-axis: Change (fold difference) at each time point is expressed as the relative difference in expression compared with the case selected as reference (relative expression level 1).

Both the tolerant cultivar Petkus and the sensitive inbred line Riodeva had higher levels of *ScCS4* transcripts in their roots than that in their leaves with Al (+) and without Al (−) after 24 h of treatment. This disparity was most noticeable and significant in the roots of plants that had not been exposed to Al (Figure 3A). Petkus roots have 50 times more *ScCS4* transcripts than leaves in the absence of Al during 24 h (−). However, Petkus roots have about 7.1-fold more *ScCS4* transcripts than the leaves after being exposed to Al for 24 h (+) (Figure 3A). Similar findings have been observed for the roots of Riodeva inbred line. Without Al, the roots of Riodeva have almost 4.7-fold more *ScCS4* transcripts than the leaves. Instead, Riodeva roots have 2.2-fold more *ScCS4* transcripts than leaves in the presence of Al (+) (Figure 3A). Moreover, *ScCS4* expression was seven times higher in the roots of Petkus than those of Riodeva without Al (−) (Figure 3B). Roots treated with Al for 24 h (+) obtained similar findings: the *ScCS4* expression was 3.4-fold higher in Petkus than in Riodeva (Figure 3B). This higher amount of mitochondrial *CS* transcripts in Petkus than in Riodeva could explain part of the Al tolerance of Petkus. However, without Al, the *ScCS4* expression levels in leaves were similar in Petkus and Riodeva (Figure 3B). With Al, the expression level in Riodeva leaves is slightly higher than that in Petkus (Figure 3B). *ScCS4* transcription in roots was diminished after exposure to Al in both Petkus and Riodeva, with higher gene repression in Petkus than Riodeva, according to time-course studies (Figures 4A,B). Furthermore, *ScCS4* transcription in roots decreased following 24 h of exposure to various Al doses (25, 50, 100, and 300 μM) (Figures 4C,D).

**FIGURE 5:**
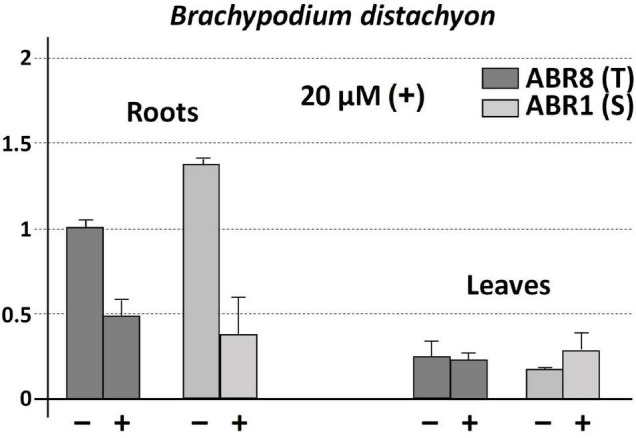
Expression patterns (qRT-PCR) of *BdCS4* at 24 h with 20 μM AlK (SO4)_2_ (+) and without AlK (SO4)_2_ (−) treatments using root apices and leaf of ABR1 (sensitive line) and ABR8 (tolerant line) of *B. distachyon*. The expression level of 18S rRNA was used as a loading control. The change at each treatment is expressed as the relative difference in expression compared with the tolerant genotype diploid (ABR8) without exposure to Al (−). Error bars represent standard deviations (SDs). All data are the means of three independent biological and technical replicates.

On the contrary, the levels of *BdCS4* transcripts were consistently higher in roots than leaves of both the resistant (ABR8) and sensitive (ABR1) *Brachypodium* lines, in the absence of Al (−) and in the presence of Al for 24 h (+) (Figure 5). The *BdCS4* expression level in the sensitive ABR1 roots without Al was slightly higher in comparison with that of the tolerant ABR8 (Figure 5). mRNA transcript levels of *ScCS4* in Petkus roots with Al were like that found for *BdCS4* in both *Brachypodium* lines (Figure 5). The *BdCS4* transcription level in roots reduced upon exposure to Al in both the tolerant ABR8 and sensitive ABR1 lines, with higher gene repression in ABR1 than in ABR8 (Figure 5). However, both lines with and without Al have similar levels of transcription in their leaves.

## Discussion

The study of identity and similarity of cDNA sequences of the hypothetical protein encoded by them (Supplementary Data 1, 1A, 2) and phylogenetic relationships indicated that the sequences isolated and characterized in rye are orthologous to the barley sequence used to design the primers. Furthermore, the isolated exon and intron structures (Figure 1A) match the structure expected in most cases for the orthologous gene of *B. distachyon*. Moreover, ScCS4 was predicted to be a mitochondrial protein. This result is consistent with that expected for mitochondrial CS proteins of the Poaceae.

Recently, advances in rye genomics have achieved the sequencing of two genomes of *S. cereale* L. (Bauer et al., 2017; Li et al., 2021). In the rye genome sequence database,^4^ we have found a cDNA and a protein sequence (SECCE6Rv1G0388260.1) that have 100 and 99.789% identity with our Imperial 2 and Ailes 1 sequences, respectively. Therefore, the sequences isolated in our different rye cultivars match the sequence of the rye genome database.

Our *ScCS4* chromosomal location on chromosome arm *6R*L (Figure 1B) agrees with the previously mentioned syntenic relationships between wheat, barley, rice, *Brachypodium*, and rye (Naranjo et al., 1987; Devos, 2005; The International Brachypodium Initiative, 2010; Martis et al., 2013). As a result, our data support the idea that the *ScCS4* gene isolated from rye is orthologous to the *T. aestivum* genes *TaCS4* (*TaCS4-A, TaCS4-B*, and *Ta CS4-D*), *HvCS4* (*H. vulgare*), *OsCS4* (*O. sativa*), and *BdCS4* (*B. distachyon*) (Figure 2 and Supplementary Figure 2). In addition, the Al tolerance gene *Alt1* has been located on chromosome *6R* in *S. cereale* L. (Gallego and Benito, 1997), and the *ScMATE3* gene, probably involved in Al detoxification mechanisms, is located on the same chromosome (Santos et al., 2020).

The *ScCS4* gene is located on a different chromosome (*6R*) than the two tolerance genes that have been detected in our crosses between sensitive and tolerant rye plants in previous publications (Fontecha et al., 2007; Silva-Navas et al., 2012). These two candidate tolerance genes, namely, *ScALMT1* and *ScMATE1*, are on the chromosome arm *7R*S (Fontecha et al., 2007; Collins et al., 2008; Silva-Navas et al., 2012).

Genetic diversity studies (Supplementary Tables 1, 2) in the *Secale* genus are essential to better characterize its genome for breeding purposes and even within *S. cereale*. This genus is one of the most tolerant to Al stress (Santos et al., 2018). All the estimated diversity parameters, including nucleotide diversity, were always higher between Poaceae species than between *S. cereale*. Our results indicated that the studied gene is highly conserved during evolution. The same is observed for other genes that encode the tricarboxylic acid cycle enzymes (Abd El-Moneim et al., 2015). This could explain the high degree of conservation detected among Poaceae species, which was even higher among rye samples. However, Santos et al. (2016, 2018) found significant genetic variability among *ScMATE1* cDNA sequences within the subspecies of *S. cereale* and even higher in the whole *Secale* genus. The obtained dendrograms using cDNA nucleotide (Figure 2) and amino acid sequences (Supplementary Figure 2) from rye and *B. distachyon* are grouped in the same cluster as the *HvCS4* cDNA and the CS4 protein from barley (this last cDNA sequence was used to design the primers). These findings support the hypothesis that both the *ScCS4* gene and protein of rye isolated in this research are orthologous to the *CS4* genes and proteins of *T. aestivum*, *T. urartu*, *A. tauschii*, *H. vulgare*, *B. distachyon*, and *B. stacei*. Moreover, these findings agree with the chromosomal location results and coincide quite well with the phylogeny of the species included in our study.

In response to Al stress, many Al-tolerant species secrete organic acids from their roots. *Triticale*, *B. nap*us, *Avena sativa*, *R. sativus*, and *S. cereale* and some tolerant varieties of *T. aestivum*, *B. distachyon*, and *B. hybridum* exudate citrate and malate. Malate is released by Al-tolerant cultivars of *P. vulgaris*, *Z. mays*, *C. tora*, *A. thaliana*, and *G. max*. Citrate is exudated by the Al-tolerant cultivars of *H. vulgare*. Finally, *Fagopyrum esculentum* and *Colocasia esculenta* exudate oxalate. However, this classification is in permanent change due to the new findings in different plant species. For instance, the roots of *P. vulgaris* and *G. max* also exudate citrate in response to Al treatment (Yang et al., 2000; Rangel et al., 2010). Moreover, another essential factor that should not be forgotten is intraspecific variability. The roots of several varieties of *T. aestivum* release only malate in response to Al stress, others only citrate, and some both organic acids simultaneously (Ryan et al., 2009; Garcia-Oliveira et al., 2016, 2018). A similar situation can be expected in other plant species. In allogamous species, the cultivars are constituted by plants with different genotypes. Therefore, our ability to detect the different organic acids exuded by the roots depends largely on the quantity of varieties and/or lines analyzed of a given species. So far, the *S. cereale* cultivars and lines studied exude malate and citrate from the roots, although in different amounts. However, because rye is an allogamous species, internal variability in the cultivars is possible since the plants would have different genotypes. Therefore, there could be plants that exude only malic or citric acids and others that exude both organic acids simultaneously. Considering that, in most of the studies on organic acid exudation in rye, pools of at least 15 or 20 plants were analyzed, it may be that some of them exude only malate or only citrate and others, both organic acids. To solve this problem, it would be necessary to analyze the exudation of each plant separately.

Consequently, the possible relationship between citrate exudation by the roots in response to Al treatment and changes in the CS enzymatic activity or CS messengers’ expression could be affected.

As already indicated in the introduction of this work, the changes in the enzymatic activity of CS produced by Al stress are different in the plant species studied. The Al treatment increases CS activity in *S. cereale* (Li et al., 2000), *P. vulgaris* (Mugai et al., 2000), and *C. tora* (Yang et al., 2004), and the roots of these three species release citrate. However, the CS activity does not increase in *T. aestivum* and triticale in their sensitive and tolerant lines (Li et al., 2000; Hayes and Ma, 2003). The Al-tolerant varieties of *T. aestivum* usually exudate only malate, and some of them release citrate. Since the wheat variety used by Hayes and Ma (2003) only releases malate, it is expected that an increase in the CS enzymatic activity will not be found. In soybean, Al stress enhances *mCS* gene expression and enzyme activity of CS (Eticha et al., 2010), and their roots release citrate and, in addition, malate. Instead, the CS activity decreases after Al treatment in *C. arabica* (Ramírez-Benítez et al., 2008). Another critical factor is that all these plant species have different genes codifying CS enzymes, some of them expressed in the cytosol and one in the mitochondria. Thereby, the enzymatic activity estimated in the previously cited works is due to the sum of activities of these different CS enzymes.

A crucial issue is the citric acid exudation pattern. There are plant species whose roots are always exuding citric acid, in the absence and in the presence of Al, showing a constitutive pattern. However, in other species, citric acid’s exudation is induced after treatment with Al, showing an inducible pattern. In rye, the inbred lines Riodeva and P105 show a constitutive citrate exudation pattern (Santos et al., 2018). However, the tolerant cultivars Imperial and Petkus have an inducible pattern of citrate exudation (Abd El-Moneim et al., 2014, 2015; Sánchez-Parra et al., 2015). In addition, different species of the *Secale* genus show different citric acid exudation patterns (Santos et al., 2018). The intraspecific variability of the exudation pattern has been less studied in plant species. The allogamous cultivars of *S. cereale* have plants with different genotypes; some could show a constitutive exudation pattern, and others could show an inducible pattern. Considering this, when analyzing the citric acid exudation pattern using groups of 15–20 plants, the expected result, if there are plants of both types, could be an inducible pattern. For *B. distachyon*, roots of sensitive line ABR1 show low citrate exudation without Al, and no changes were observed by Al treatment. However, roots of the tolerant line ABR8 show a clearly Al-inducible citrate exudation pattern (Contreras et al., 2014).

Changes in *CS* gene expression due to Al treatment are also variable depending on the species or whether plants are sensitive or tolerant to Al. *CS* expression does not change significantly due to Al treatment in both *P. vulgaris* (Eticha et al., 2010) and *A. thaliana* (Kumari et al., 2008). However, overexpression of *mCS* genes led to increased citrate efflux in carrot, *A. thaliana*, and canola cells (Koyama et al., 1999, 2000; Anoop et al., 2003), respectively.

As mentioned in the introduction, overexpression of a bacterial *CS* gene in transgenic tobacco and papaya plants resulted in improved Al tolerance and citrate overproduction (de la Fuente et al., 1997). However, Delhaize et al. (2001), using transgenic tobacco plants, did not observe these results and reported that two transgenic alfalfa plants expressing CS did not show an improvement in Al tolerance. On the contrary, Barone et al. (2008) detected transgenic alfalfa plants with more Al tolerance than non-transgenic control. The rice mitochondrial gene *OsCS1* was induced by Al treatment, and several tobacco transgenic lines expressing *OsCS1* showed increased citrate efflux and high Al tolerance (Han et al., 2009). Finally, the Al-inducible expression of mitochondrial *CS* together with enhanced citrate release mediates Al resistance in the tree *P. falcataria* (Osawa and Kojima, 2006).

It is the first time that repression of the *mCS* gene following Al treatment was observed in *S. cereale* and *B. distachyon*, although repression of other genes related to organic acid metabolism (Abd El-Moneim et al., 2015) or related to Al stress (Sánchez-Parra et al., 2015) has been previously detected in rye.

This gene repression could be indirectly associated with malate and citrate exudation. When the mitochondrial genes *MDH* (malate dehydrogenase) and *FUM* (fumarase) were repressed in transgenic *Solanum lycopersicum* plants, a decrease in both root length and exuded pH was observed, and the exudation of malate and citrate was activated (van der Merwe et al., 2009). Thus, increased root exudation of organic acids may be associated with decreased pH. In yeast and transgenic *B. napus*, Anoop et al. (2003) found that the regulation of several enzymes involved in citrate synthesis and turnover, such as MDH, CS, ACO (aconitase), and IDH (isocitrate dehydrogenase), could be recognized as potential targets for gene manipulation to understand better the role of citrate metabolism in mediating Al tolerance. Another vital aspect that could be related to mitochondrial CS expression is the exudation of citrate by the roots carried out by citrate-transporting membrane proteins that may or may not be activated by aluminum treatment.

At least two different genes, probably involved in root citrate exudation and Al tolerance, namely, *ScMATE1* (*ScFRLD1* or *ACCT1*) and *ScMATE2* (*ScFRLD2*), have been reported in rye (Yokosho et al., 2010; Silva-Navas et al., 2012). The *ScMATE1* is induced by Al treatment in the sensitive Riodeva inbred line (Silva-Navas et al., 2012), but not in the IR51 rye inbred line (Yokosho et al., 2010). The transcription factors *ScSTOP1* and *TaSTOP1* seem to be necessary for the expression of the genes *ScALMT1*, *ScMATE1* (from rye), *TaALMT1*, and *TaMATE1* (from wheat) (Garcia-Oliveira et al., 2013; Silva-Navas et al., 2021). The *ScMATE1* gene is Al-induced in other tolerant rye cultivars like Imperial, Petkus, and the inbred line 2,672/4. However, Al stress does not induce this gene in Ailés tolerant rye cultivar. The tolerance of the Ailés cultivar is mainly due to the presence of an allele of the *ScALMT1* gene that codes for a malate transporter activated by Al (Fontecha et al., 2007; Collins et al., 2008). The *ScMATE1* allele present in the sensitive inbred line Riodeva is a tolerant allele (Silva-Navas et al., 2012). This contradictory finding is explained because the roots of the inbred line Riodeva can grow at concentrations between 100 and 150 μM. Therefore, inbred line Riodeva compared with other rye cultivars and lines whose roots grow at concentrations higher than 300 μM is a sensitive rye. However, Riodeva is tolerant compared with other plant species such as *H. vulgare*, *T. aestivum*, *T. turgidum*, *A. thaliana*, and *B. distachyon* whose roots do not grow at concentrations higher than 100 μM.

Repression of *ScCS4* and *BdCS4* mRNA transcription in response to Al in *S. cereale* and *B. distachyon* roots indicates that manipulation of mitochondrial *CS* gene expression can enhance Al tolerance in rye and *B. distachyon* and that overexpression of these genes in sensitive lines can also improve their tolerance.

## Data Availability Statement

The original contributions presented in the study are included in the article/Supplementary Material, further inquiries can be directed to the corresponding author/s.

## Author Contributions

CB designed and performed this research. DAE-M and RC obtained the rye CS sequences and the expression data. FG and JS-N analyzed the sequences and carried out the phylogenetic analyses. AF revised this draft by rewriting the discussion and commenting. All authors commented on the manuscript.

## Conflict of Interest

The authors declare that the research was conducted in the absence of any commercial or financial relationships that could be construed as a potential conflict of interest.

## Publisher’s Note

All claims expressed in this article are solely those of the authors and do not necessarily represent those of their affiliated organizations, or those of the publisher, the editors and the reviewers. Any product that may be evaluated in this article, or claim that may be made by its manufacturer, is not guaranteed or endorsed by the publisher.

## Footnotes

1
http://bioinf.cs.ucl.ac.uk/psipred/

2
www.jmol.org

3
http://provean.jcvi.org/index.php

4
https://galaxy-web.ipk-gatersleben.de/

## Supplementary Material

Supplementary Figure 1 On the left, the hypothetical ScCS protein secondary structure was obtained with PSIPRED v3.0 (http://bioinf.cs.ucl.ac.uk/psipred/). The secondary structure is made up of 23 α-helix regions and 3 β-sheet regions. On the right, the hypothetical tertiary structure of ScCS from the cultivars Ailés and Petkus. The tertiary structure was obtained with the SWISS-MODEL program. The protein from the Protein DataBase (PDB) showed that the greatest similarity to ours (83.3%) was from *Arabidopsis thaliana* (with a resolution of 2.00 Å).

Supplementary Figure 2 The dendrogram was obtained from the sequences of amino acids of the hypothetical ScCS4 protein and CS proteins of various species of Poaceae and other eudicots. The studied sequences of proteins were extracted from NCBI and Phytozome. Bootstraps with 10,000 replicates were calculated to test the robustness of the dendrogram. *Secale cereale* L. is in red color, the blue lines indicate the Poaceae species, and the red lines indicate some eudicot species.

Supplementary Data 1 Alignment of *ScCS4* cDNA sequences corresponding to eight different sequences (different clones) of *S. cereale*, one from cv. Ailés, four from cv. Imperial, two from cv. Petkus, and the last from inbred line Riodeva.

Supplementary Data 1A Alignment of *ScCS4* amino acid sequences corresponding to eight different sequences (different clones) of *S. cereale*, one from cv. Ailés, four from cv. Imperial, two from cv. Petkus, and the last from inbred line Riodeva.

Supplementary Data 2 Alignment of *ScCS4* cDNA sequence corresponding to *S. cereale* cv. Ailés with those found in other six species of Poaceae.

Supplementary Data 3 Alignment of ScCS4 putative protein sequences corresponding to the rye cultivars Ailés, Imperial, and Petkus, and the inbred line Riodeva.

Supplementary Data 4 Alignment of ScCS4 putative protein sequences corresponding to *S. cereale* cv. Imperial, several Poaceae species, and other plant species.
