# Supplementary figures and images for: Repression of Mitochondrial Citrate Synthase Genes by Aluminum Stress in Roots of Secale cereale and Brachypodium distachyon
Source: Front Plant Sci. 2022 Apr 7;13:832981. doi: 10.3389/fpls.2022.832981 (PMC9021840; doi:10.3389/fpls.2022.832981)

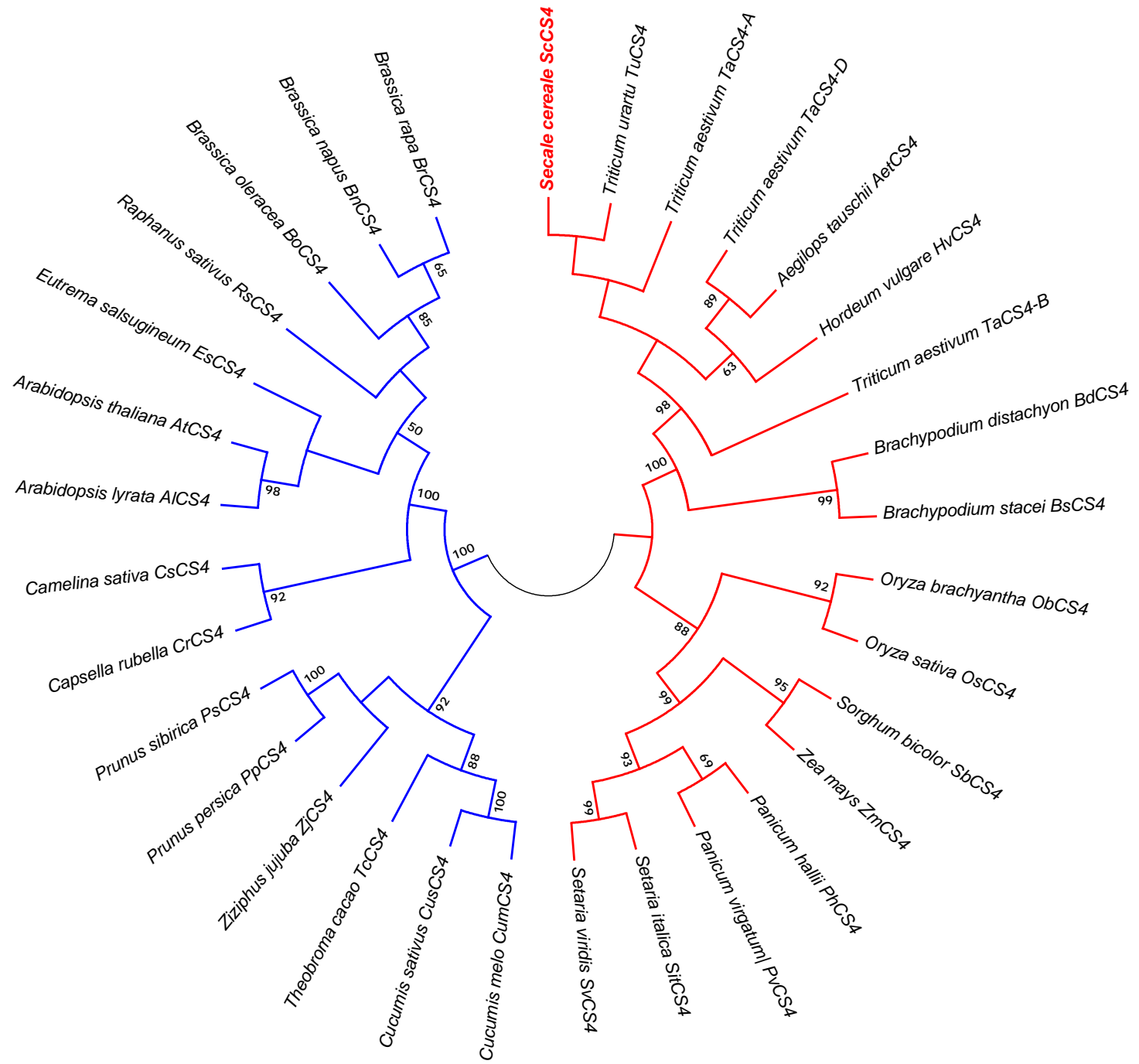

Supplement: Supplementary Figure 1 — On the left, the hypothetical ScCS protein secondary structure was obtained with PSIPRED v3.0 (http://bioinf.cs.ucl.ac.uk/psipred/). The secondary structure is made up of 23 α-helix regions and 3 β-sheet regions. On the right, the hypothetical tertiary structure of ScCS from the cultivars Ailés and Petkus. The tertiary structure was obtained with the SWISS-MODEL program. The protein from the Protein DataBase (PDB) showed that the greatest similarity to ours (83.3%) was from Arabidopsis thaliana (with a resolution of 2.00 Å). [file Image_1.pdf]

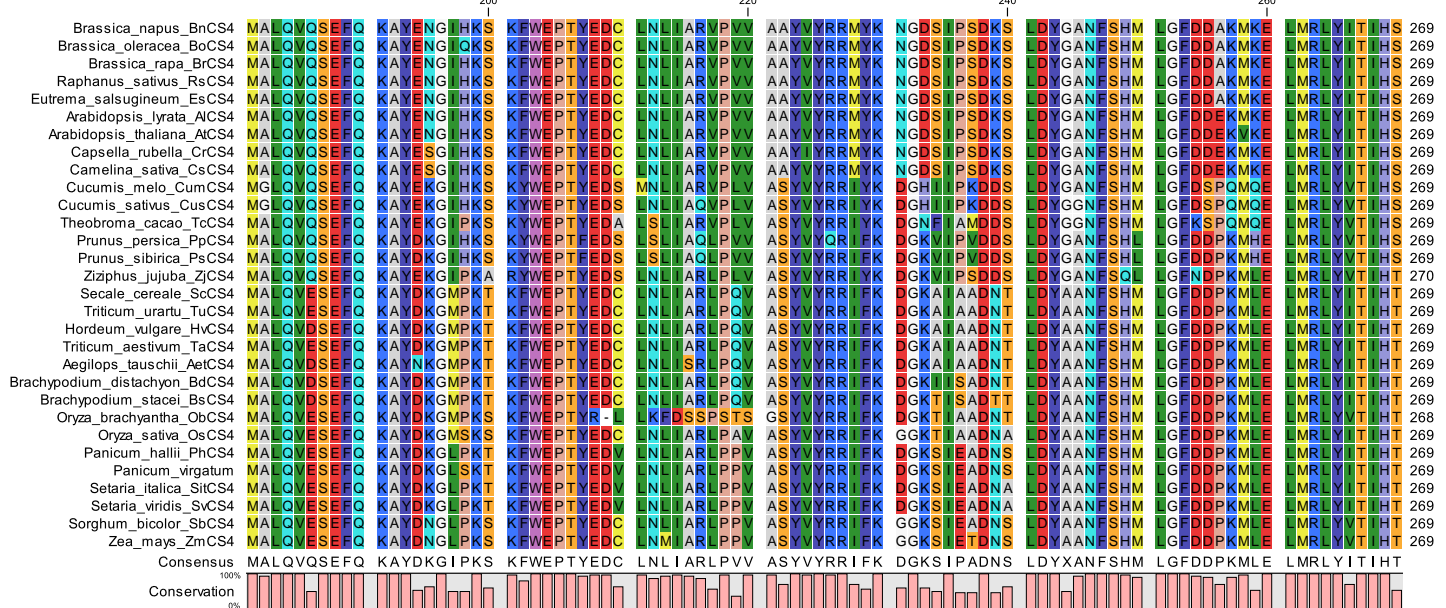

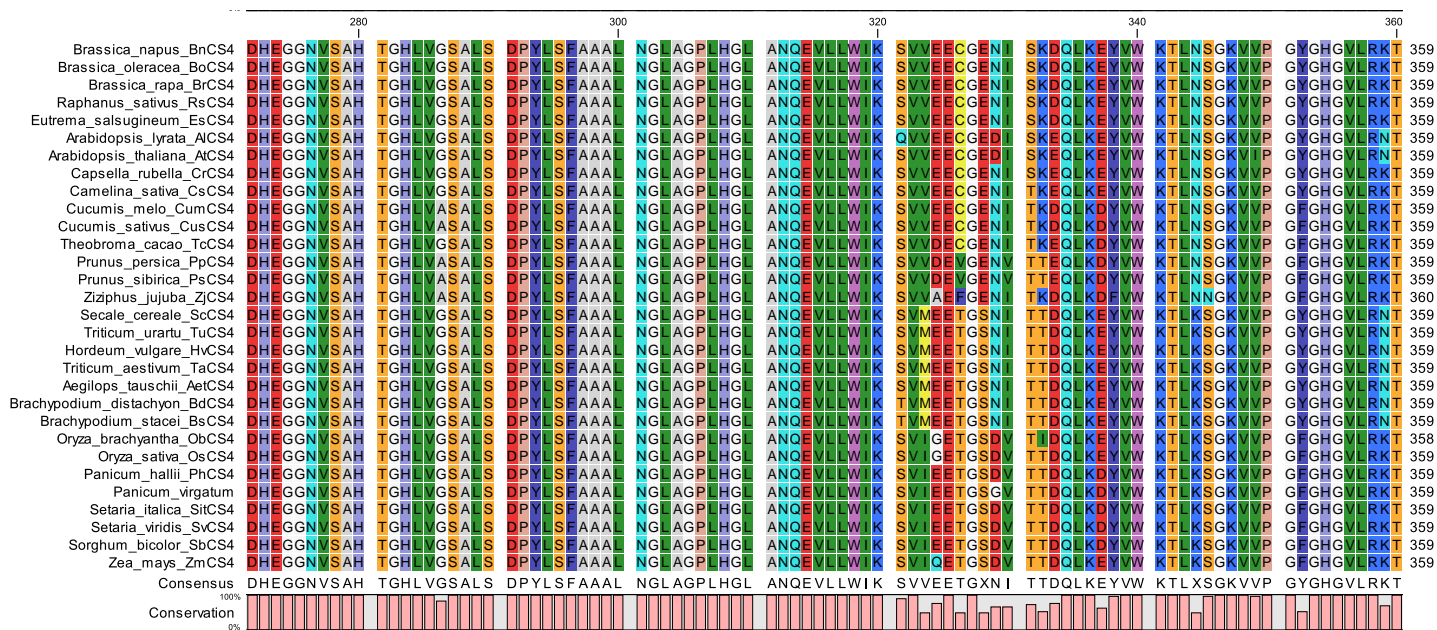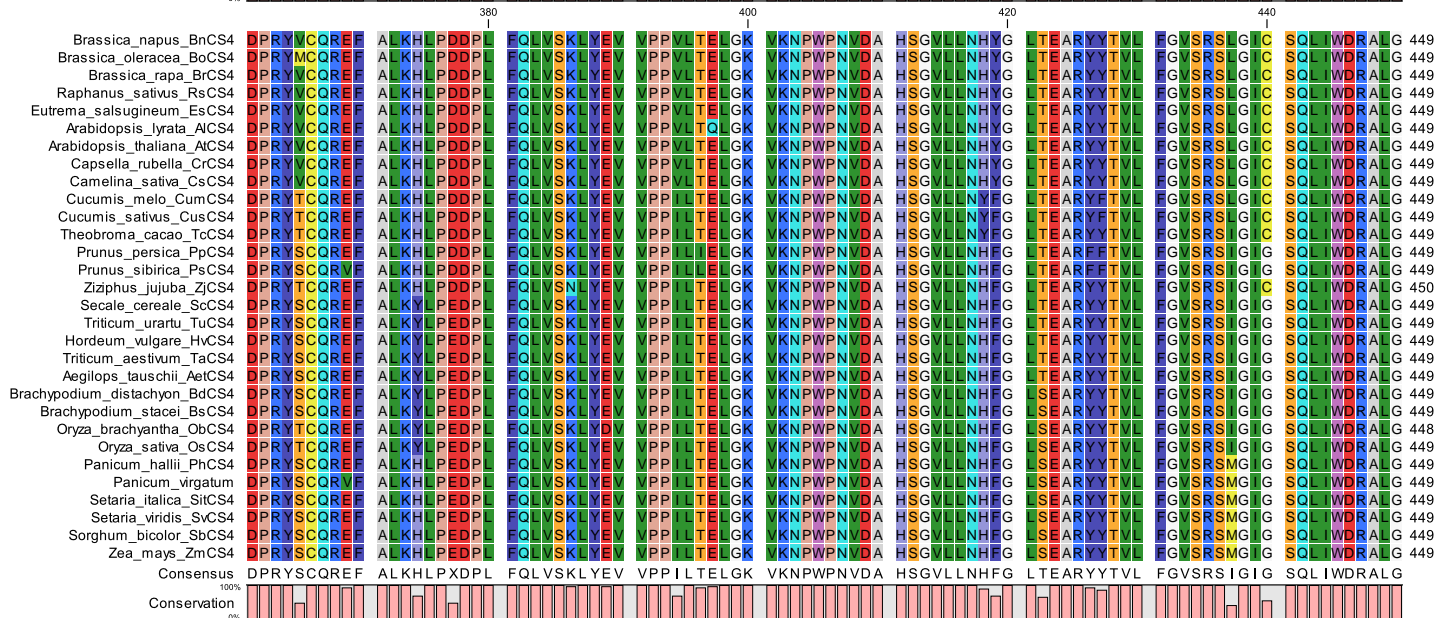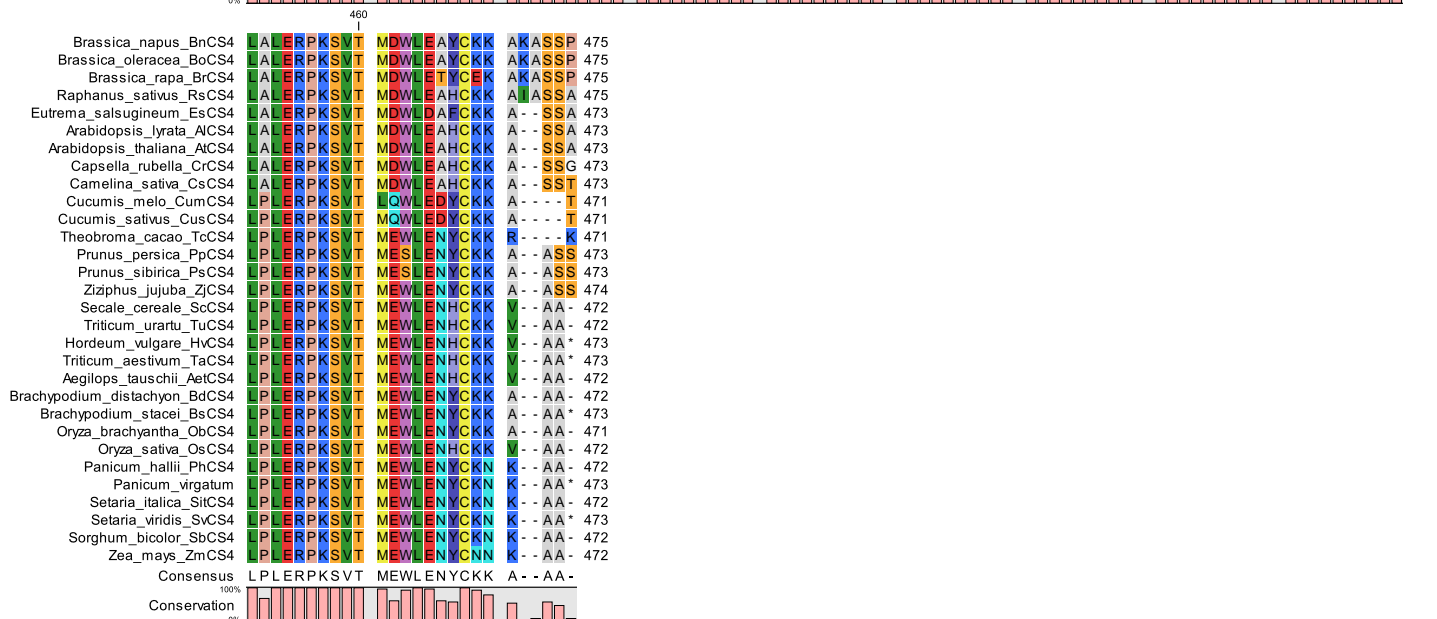

Supplement: Supplementary Data 1A — Alignment of ScCS4 amino acid sequences corresponding to eight different sequences (different clones) of S. cereale, one from cv. Ailés, four from cv. Imperial, two from cv. Petkus, and the last from inbred line Riodeva. [file Data_Sheet_5.pdf]

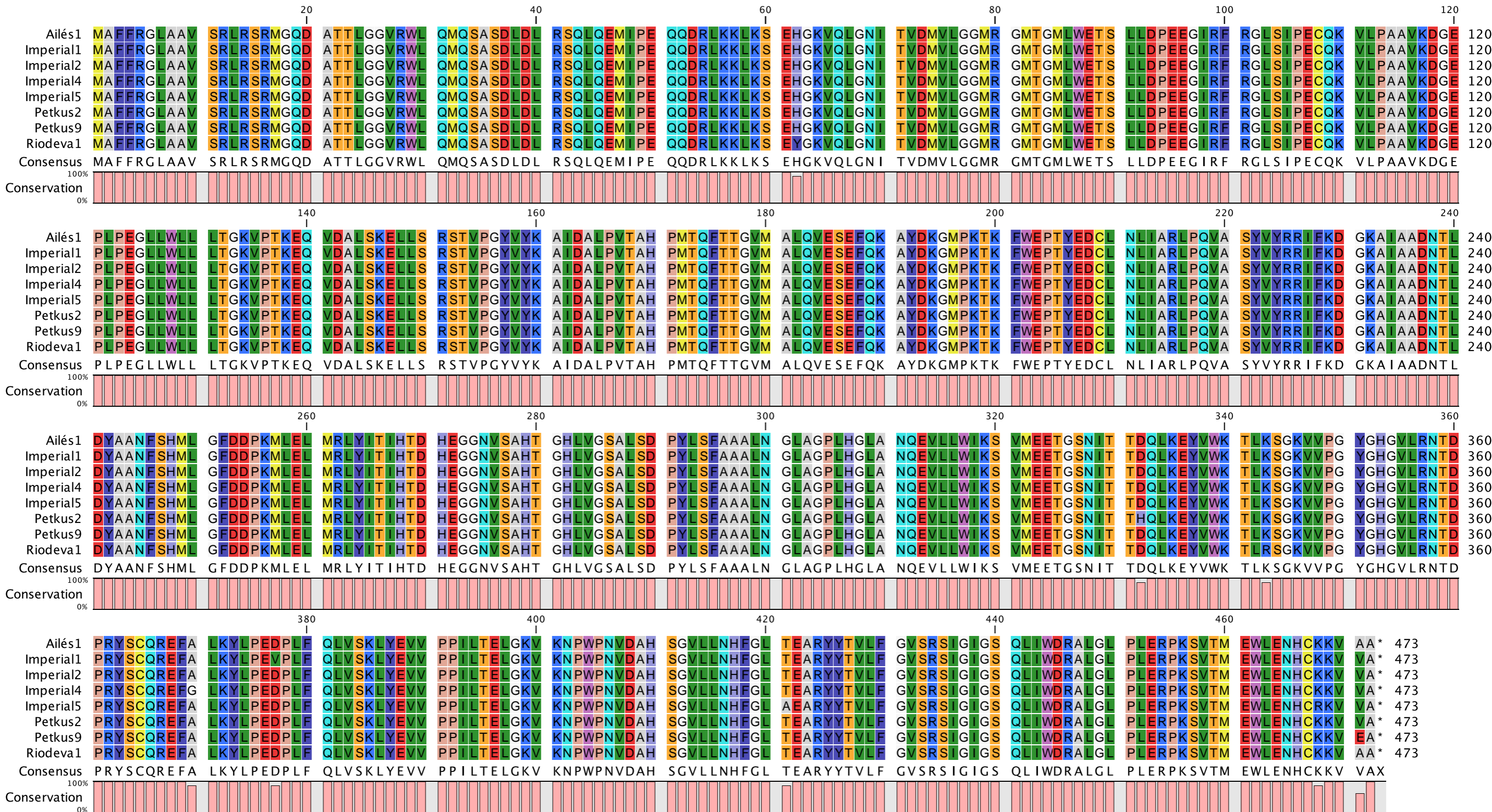

Supplement: Supplementary Data 2 — Alignment of ScCS4 cDNA sequence corresponding to S. cereale cv. Ailés with those found in other six species of Poaceae. [file Data_Sheet_2.pdf]

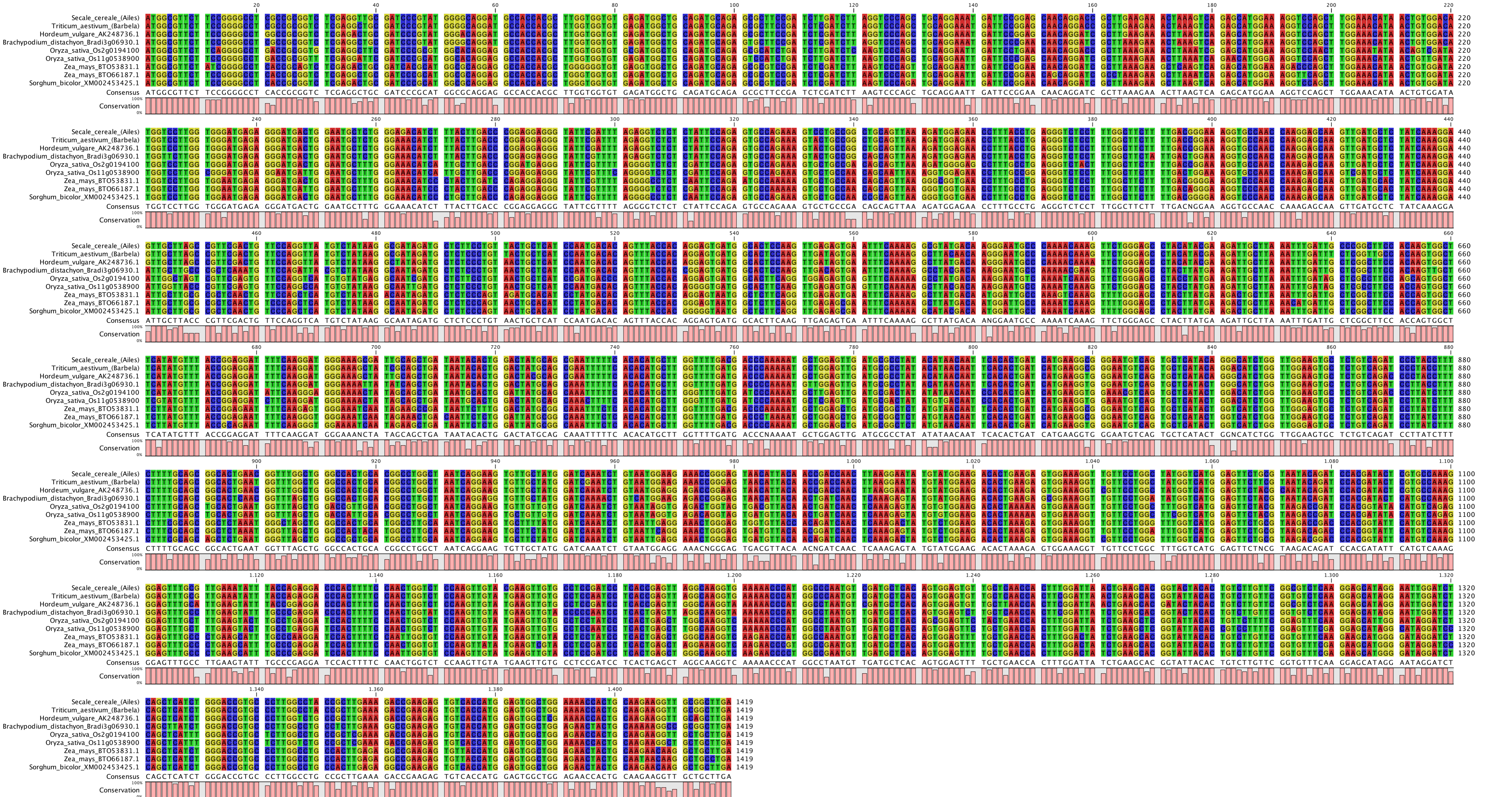

Supplement: Supplementary Data 3 — Alignment of ScCS4 putative protein sequences corresponding to the rye cultivars Ailés, Imperial, and Petkus, and the inbred line Riodeva. [file Data_Sheet_3.pdf]

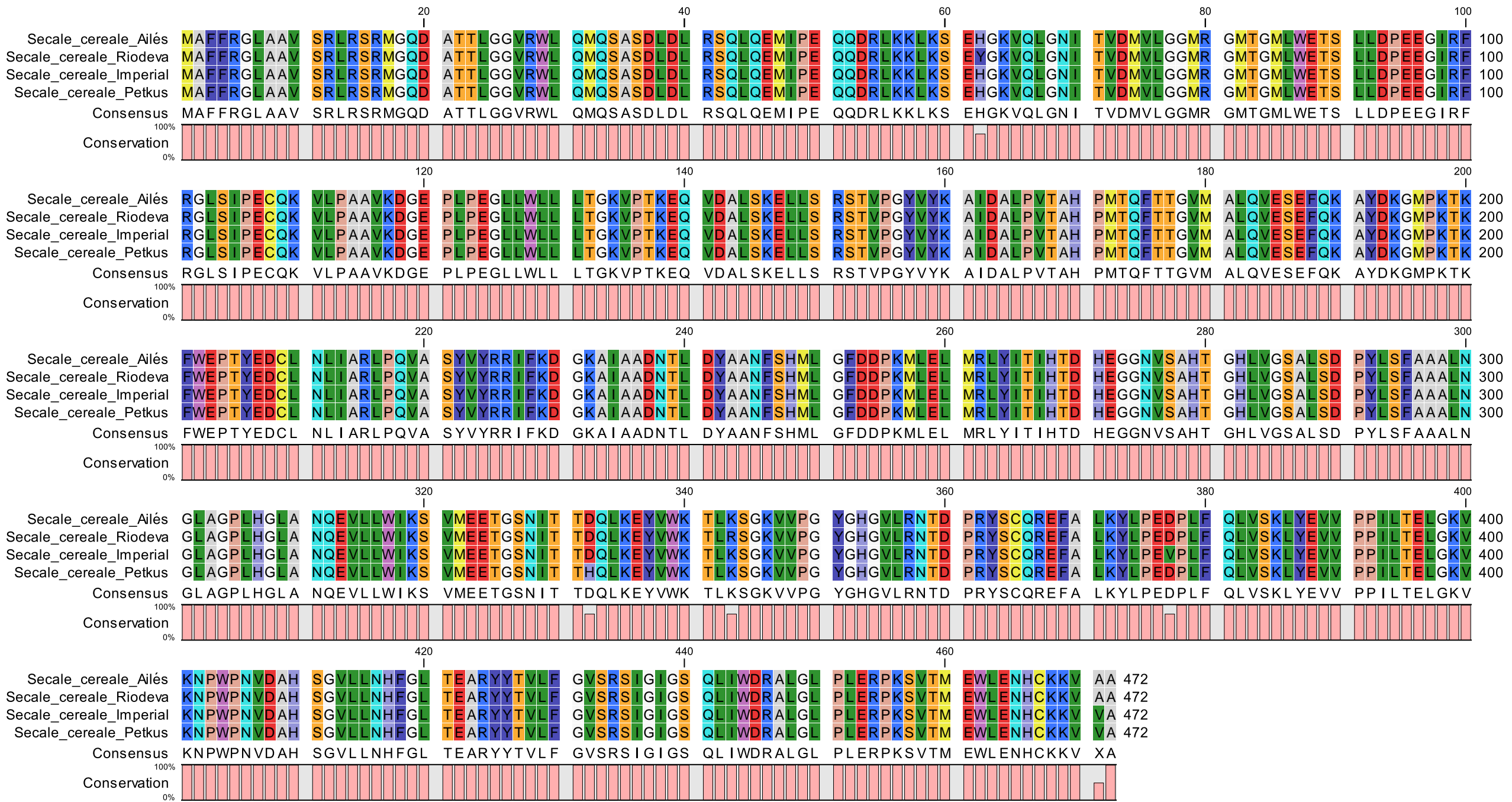

Supplement: Supplementary Data 4 — Alignment of ScCS4 putative protein sequences corresponding to S. cereale cv. Imperial, several Poaceae species, and other plant species. [file Data_Sheet_4.pdf]
