# Supplementary material for: Repression of Mitochondrial Citrate Synthase Genes by Aluminum Stress in Roots of Secale cereale and Brachypodium distachyon
Source: Front Plant Sci. 2022 Apr 7;13:832981. doi: 10.3389/fpls.2022.832981 (PMC9021840; doi:10.3389/fpls.2022.832981)
Supplement: Supplementary Figure 2 — The dendrogram was obtained from the sequences of amino acids of the hypothetical ScCS4 protein and CS proteins of various species of Poaceae and other eudicots. The studied sequences of proteins were extracted from NCBI and Phytozome. Bootstraps with 10,000 replicates were calculated to test the robustness of the dendrogram. Secale cereale L. is in red color, the blue lines indicate the Poaceae species, and the red lines indicate some eudicot species. [file Presentation_1.pptx]

## Slide 1
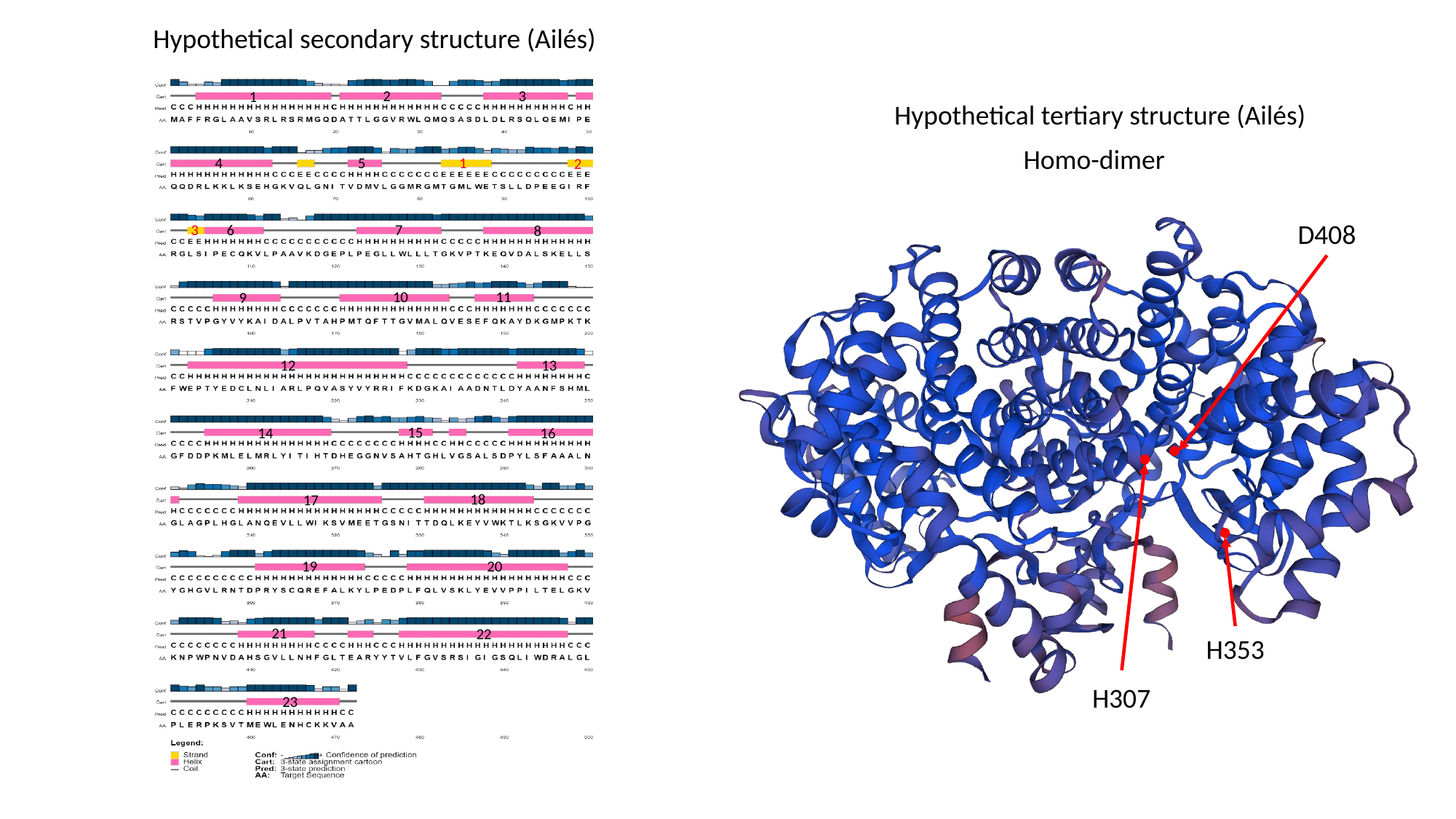

Hypothetical secondary structure (Ailés)
3
2
1
Hypothetical tertiary structure (Ailés)
Homo-dimer
5
1
4
2
D408
H353
H307
6
7
3
8
11
10
9
13
12
15
14
16
18
17
20
19
21
22
23

## Slide 2
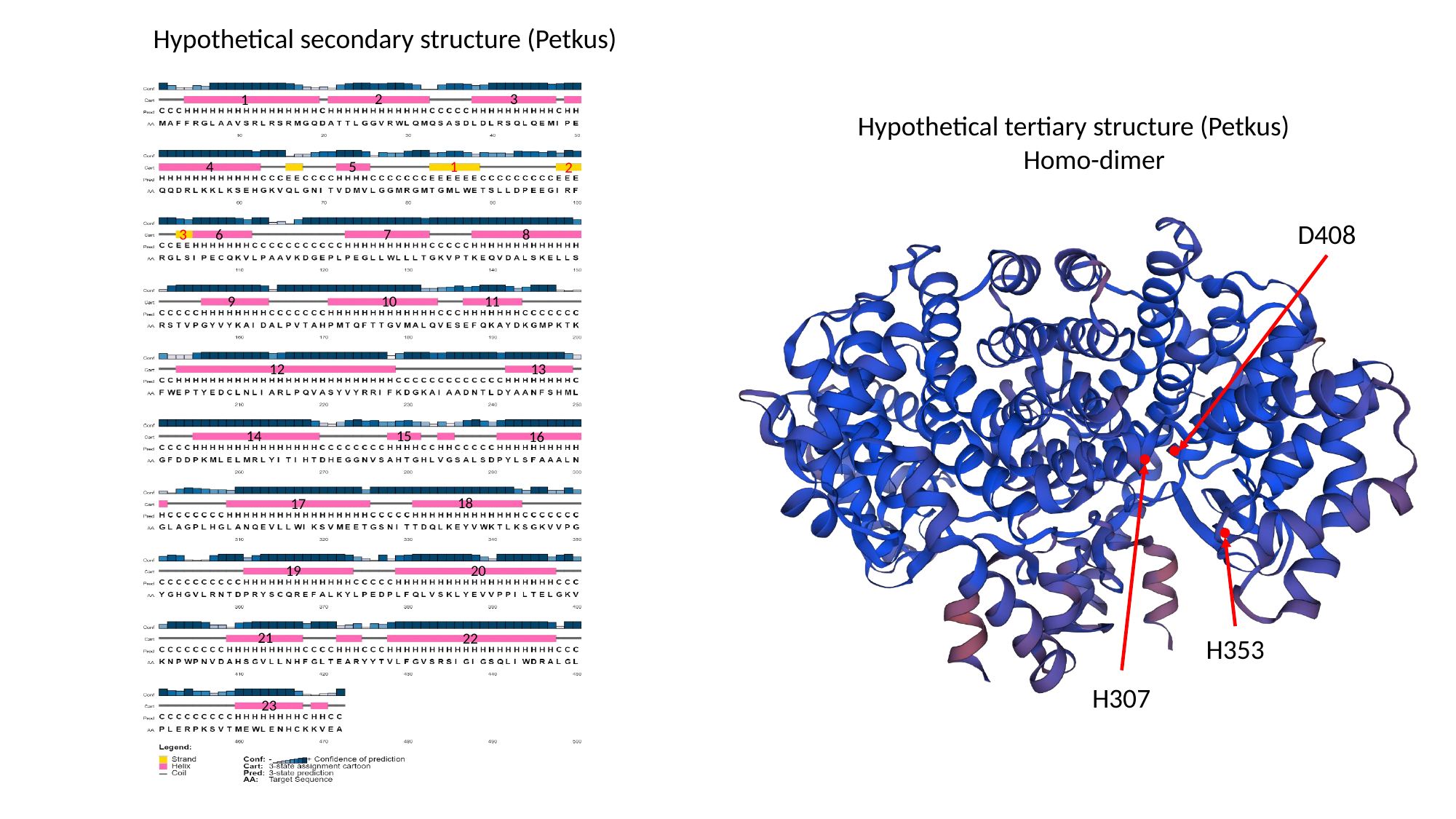

Hypothetical secondary structure (Petkus)
3
2
1
Hypothetical tertiary structure (Petkus)
Homo-dimer
5
1
4
2
D408
H353
H307
6
7
3
8
11
10
9
13
12
15
14
16
18
17
20
19
21
22
23

## Slide 3
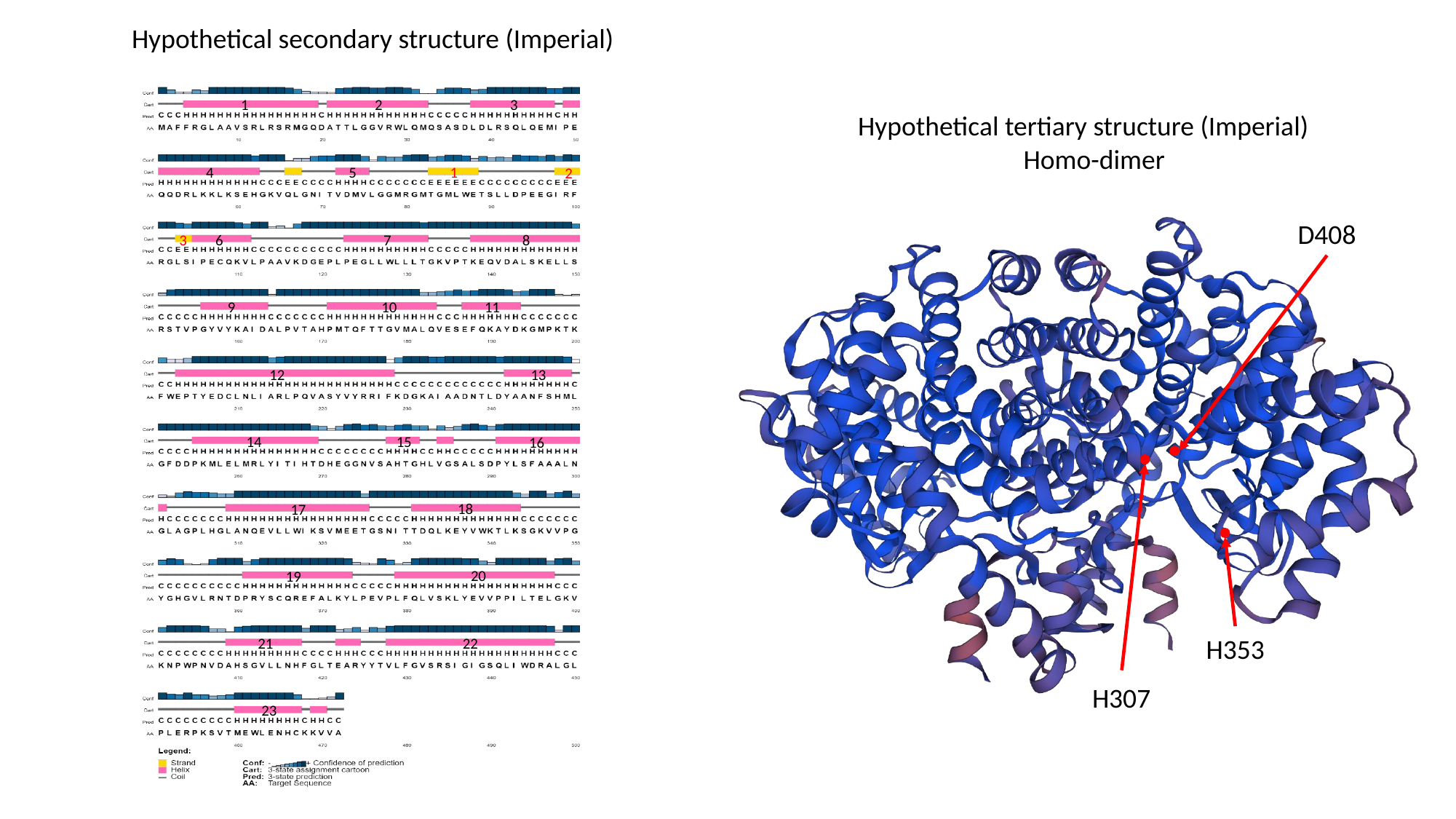

Hypothetical secondary structure (Imperial)
3
2
1
Hypothetical tertiary structure (Imperial)
Homo-dimer
5
1
4
2
D408
H353
H307
6
7
3
8
11
10
9
13
12
15
14
16
18
17
20
19
21
22
23

## Slide 4
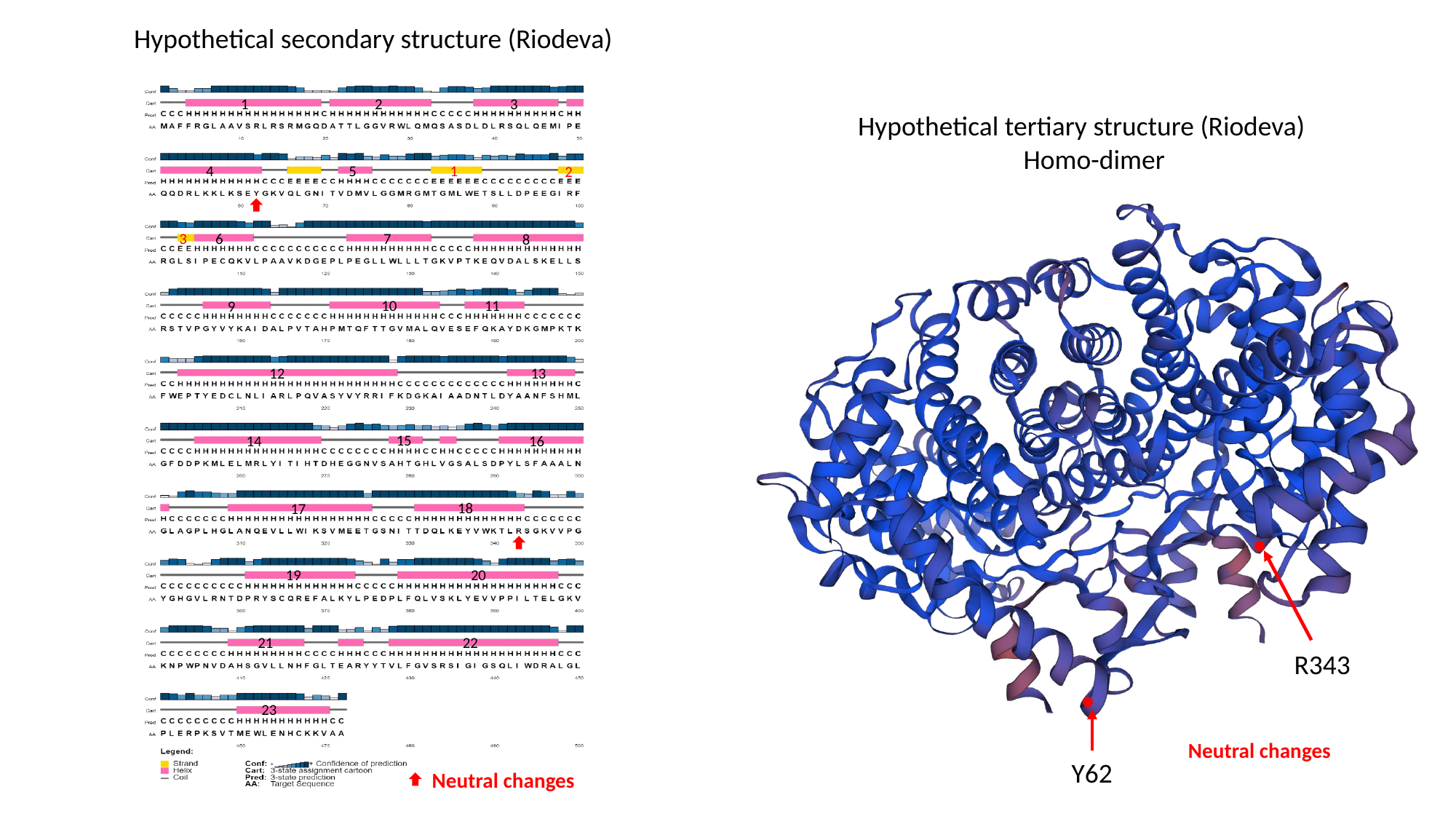

Hypothetical secondary structure (Riodeva)
3
2
1
Hypothetical tertiary structure (Riodeva)
Homo-dimer
5
1
4
2
6
7
3
8
11
10
9
13
12
15
14
16
18
17
20
19
21
22
R343
23
Neutral changes
Y62
Neutral changes
