# Supplementary material for: Repression of Mitochondrial Citrate Synthase Genes by Aluminum Stress in Roots of Secale cereale and Brachypodium distachyon
Source: Front Plant Sci. 2022 Apr 7;13:832981. doi: 10.3389/fpls.2022.832981 (PMC9021840; doi:10.3389/fpls.2022.832981)
Supplement: Supplementary file 8 [file Table_1.docx]

**Supplementary Table S1.** Genetic variability analysis in cDNAs sequences (exons 1 to 19) of *ScCS* among eight rye sequences: Ailés (one), Imperial (four), Petkus (two), and Riodeva (one) (see Supplementary Data S1).

| Rye | 1 | 2 | 3 | 4 | 5 | 6 | 7 | 8 | 9 | 10 | 11 | 12 | 13 | 14 | 15 | 16 | 17 | 18 | 19 | cDNA |
| --- | --- | --- | --- | --- | --- | --- | --- | --- | --- | --- | --- | --- | --- | --- | --- | --- | --- | --- | --- | --- |
| NS | 51 | 63 | 42 | 66 | 66 | 114 | 64 | 83 | 50 | 78 | 129 | 43 | 87 | 102 | 108 | 51 | 74 | 52 | 96 | 1419 |
| INDEL | 0 | 0 | 0 | 0 | 0 | 0 | 0 | 0 | 0 | 0 | 0 | 0 | 0 | 0 | 0 | 0 | 0 | 0 | 0 | 0 |
| IMS | 51 | 63 | 42 | 65 | 65 | 114 | 64 | 83 | 50 | 78 | 127 | 43 | 87 | 100 | 106 | 51 | 72 | 52 | 94 | 1407 |
| VPS (SNPs) | 0 | 0 | 0 | 1 | 1 | 0 | 0 | 0 | 0 | 0 | 2 | 0 | 0 | 2 | 2 | 0 | 2 | 0 | 2 | 12 |
| SVS | 0 | 0 | 0 | 1 | 1 | 0 | 0 | 0 | 0 | 0 | 1 | 0 | 0 | 2 | 2 | 0 | 2 | 0 | 1 | 10 |
| PIS | 0 | 0 | 0 | 0 | 0 | 0 | 0 | 0 | 0 | 0 | 1 | 0 | 0 | 0 | 0 | 0 | 0 | 0 | 1 | 2 |
| Total No  of SC | 0 | 0 | 0 | 0 | 1 | 0 | 0 | 0 | 0 | 0 | 2 | 0 | 0 | 0 | 0 | 0 | 1 | 0 | 0 | 4 |
| Total No  of NSC | 0 | 0 | 0 | 1 | 0 | 0 | 0 | 0 | 0 | 0 | 0 | 0 | 0 | 2 | 2 | 0 | 1 | 0 | 3 | 9 |

**NS**: Number of Sites, **INDEL**: Insertions Deletions polymorphism, **IMS**: Invariable Monomorphic Sites, **VPS**: Variable Polymorphic Sites, **SNP**: Single Nucleotide Polymorphism, **SVS**: Singleton Variable Sites, **PIS**: Parsimony Informative Sites, **CS**: Conservation Sequence, **Total No** **SC**: Total number of Synonymous Changes, **Total No** **NSC**: Total number of Non-Synonymous Changes.

**Supplementary Table S2.** Genetic variability analysis in cDNAs sequences (exons 1 to 19) of *CS* gene among seven species of Poaceae, including rye (a total of 9 sequences) (see Supplementary Data S2).

| Poaceae | 1 | 2 | 3 | 4 | 5 | 6 | 7 | 8 | 9 | 10 | 11 | 12 | 13 | 14 | 15 | 16 | 17 | 18 | 19 | cDNA |
| --- | --- | --- | --- | --- | --- | --- | --- | --- | --- | --- | --- | --- | --- | --- | --- | --- | --- | --- | --- | --- |
| NS | 51 | 63 | 42 | 66 | 66 | 114 | 64 | 83 | 50 | 78 | 129 | 43 | 87 | 102 | 108 | 51 | 74 | 52 | 96 | 1419 |
| INDEL | 0 | 0 | 0 | 0 | 0 | 0 | 0 | 0 | 0 | 0 | 0 | 0 | 0 | 0 | 0 | 0 | 0 | 0 | 0 | 0 |
| IMS | 38 | 51 | 35 | 49 | 53 | 91 | 50 | 60 | 35 | 60 | 92 | 38 | 68 | 73 | 83 | 40 | 60 | 38 | 76 | 1090 |
| VPS (SNPs) | 13 | 12 | 7 | 17 | 13 | 23 | 14 | 23 | 15 | 18 | 37 | 5 | 19 | 29 | 25 | 11 | 14 | 14 | 20 | 329 |
| SNPs/100 | 25 | 19 | 17 | 26 | 20 | 20 | 22 | 28 | 30 | 23 | 29 | 12 | 22 | 28 | 23 | 21 | 19 | 27 | 21 | 23 |
| SVS | 8 | 4 | 2 | 10 | 6 | 8 | 2 | 8 | 5 | 10 | 15 | 1 | 2 | 8 | 3 | 5 | 3 | 3 | 4 | 107 |
| PIS | 5 | 8 | 5 | 7 | 7 | 15 | 12 | 15 | 10 | 8 | 22 | 4 | 17 | 21 | 22 | 6 | 11 | 11 | 16 | 222 |
| Total No  of SC | 9 | 8 | 6 | 16 | 12 | 24 | 8 | 18 | 13 | 16 | 28 | 6 | 21 | 25 | 27 | 11 | 19 | 16 | 17 | 300 |
| Total No  of NSC | 3 | 2 | 3 | 0 | 2 | 2 | 4 | 4 | 6 | 4 | 6 | 0 | 0 | 5 | 5 | 0 | 1 | 1 | 3 | 51 |

**NS**: Number of Sites, **INDEL**: Insertions Deletions polymorphism, **IMS**: Invariable Monomorphic Sites, **VPS**: Variable Polymorphic Sites, **SNP**: Single Nucleotide Polymorphism, **SVS**: Singleton Variable Sites, **PIS**: Parsimony Informative Sites, **CS**: Conservation Sequence, **Total No** **SC**: Total number of Synonymous Changes, **Total No** **NSC**: Total number of Non-Synonymous Changes.

**Supplementary Table S3.** Genetic variability comparison between cDNAs of *ScCS* gene in different cultivars of rye and different species of Poaceae (including rye).

| *ScCS* | TNS | NS | INDEL | IMS | VPS (SNPs) | SVS | PIS | NH | DH | π | CS | Total No SC | Total No NSC. |
| --- | --- | --- | --- | --- | --- | --- | --- | --- | --- | --- | --- | --- | --- |
| Rye | 8 | 1419 | 0 | 1407 | 12 | 10 | 2 | 8 | 1 | 0.00269 | 0.992 | 4 | 9 |
| Poaceae | 9 | 1419 | 0 | 1090 | 329 | 107 | 222 | 9 | 1 | 0.10097 | 0.768 | 300 | 51 |

**TNS**: Number of sequences, **NS**: Number of Sites, **INDEL**: Insertions Deletions polymorphism, **IMS**: Invariable Monomorphic Sites, **VPS**: Variable Polymorphic Sites, **SVS**: Singleton Variable Sites, **PIS**: Parsimony Informative Sites, **NH**: Number of Haplotypes, **DH**: Haplotype Diversity, **π**: Nucleotide Diversity, **CS**: Conservation Sequence, **Total No** **SC**: Total number of Synonymous Changes, **Total No** **NSC**: Total number of Non Synonymous Changes.
